# Supplementary material for: Associations between Early-Life Food Deprivation and Risk of Frailty of Middle-Age and Elderly People: Evidence from the China Health and Retirement Longitudinal Study
Source: Nutrients. 2021 Aug 31;13(9):3066. doi: 10.3390/nu13093066 (PMC8472025; doi:10.3390/nu13093066)
Supplement: Supplementary file 1 [file nutrients-13-03066-s001.zip › nutrients-1316230-supplementary.pdf]

Table S1. Characteristics of the participants experienced hungry

Variable	Self-reported starvation	Extreme food deprivation
	6986	1490
Age, years (SD)	61.3(9.5)	63.1(8.7)
Gender		
Man	3386	585
Women	3600	805
Hungry period		
Age 0-6	4158	693
Age 6-12	6171	1025
Frailty		
Non-frail	3789	708
Pre-frail	2836	671
Frail	361	111
Marital		
Married	5809	1179
Divorce	366	84
Widowed	757	213
Never married	54	14
Living area		
Urban	2323	414
Rural	4663	1075
Smoking	3172	637
drinking	1862	391

Table S2 Association between childhood food deprivation and physical (pre)frailty

Variable	Model1<br>OR (95% CI)	Model2<br>OR (95% CI)	Model3<br>OR (95% CI)
Age, per 10 years	1.45(1.42-1.48) **	1.33(1.3-1.36) **	1.29(1.27-1.32) **
Gender[a]	1.8 (1.73-1.87) **	1.52(1.46-1.58) **	1.53(1.45-1.62) **
Food deprivation[b]			
Moderately deprivation	1.33(1.28-1.39) **	1.26(1.21-1.31) **	1.23(1.18-1.28) **
Extreme deprivation	1.94(1.82-2.08) **	1.70(1.59-1.82) **	1.67(1.56-1.78) **
Education[c]			
Can read and write		0.96(0.91-1.02)	0.94(0.89-0.99)
Primary school		0.76(0.72-0.81) **	0.73(0.7-0.77) **
Junior school		0.59(0.55-0.62) **	0.55(0.52-0.59) **
High school and above		0.37(0.35-0.4) **	0.35(0.33-0.38) **
Marita[d]			
Divorce		1.3(1.2-1.41) **	1.31(1.21-1.42) **
Widowed		1.15(1.08-1.22) *	1.17(1.1-0) **
Never married		1.7(1.35-2.15) *	1.59(1.27-0) *
Living area[e]		0.67(0.65-0.7) **	0.66(0.63-0.69) **
BMI[f]			
<18.5			1.24(1.15-1.33) **
24-28			1.09(1.04-1.13) *
≥28			1.56(1.47-1.65) **
Smoking			1.29(1.22-1.36) **
drinking			0.74(0.71-0.78) **

Note: OR, odds ratio; CI; confidence interval;

[a]Gender, reference male.

[b]Food deprivation, reference category none.

[c]Education, reference illiterate.

[d]Marital, reference Married.

[e]Living area, reference rural

[f]BMI, reference normal weight, 18.5≤BMI<24

** p< 0.01 * p<0.05 <0.1

Table S3 Association between extreme food deprivation and physical (pre)frailty

Variable	Model1<br>OR (95% CI)	Model2<br>OR (95% CI)	Model3<br>OR (95% CI)
Age, per 10 yeaes	1.37 (1.33-1.41) **	1.26(1.23-1.30) **	1.25(1.22-1.29) **
Gender[a]	1.79 (1.70-1.87) **	1.52(1.44-1.60) **	1.58(1.47-1.70) **
Extreme hungry[b]	1.47 (1.38-1.57) **	1.35(1.26-1.44)	1.34(1.26-1.43) **
Education[c]			
Can read and write		0.97(0.90-1.04)	0.97(0.90-1.04)
Primary school		0.77(0.71-0.82) **	0.77(0.72-0.82) **
Junior school		0.57(0.53-0.62) **	0.57(0.53-0.61) **
High school and above		0.37(0.34-0.41) **	0.38(0.34-0.42) **
Marital[d]			
Divorce		1.39(1.26-1.53) **	1.40(1.27-1.54) **
Widowed		0.99(0.92-1.08)	0.98(0.91-1.07)
Never married		1.62(1.22-2.13) ·	1.54(1.27-2.03)
Living area[e]		0.69(0.65-0.72) **	0.67(0.64-0.71) **
BMI[f]			
<18.5			1.28(1.17-1.41) **
24-28			1.08(1.02-1.14)
≥28			1.43(1.33-1.54) **
Smoking			1.27(1.18-1.36) *
drinking			0.76(0.71-0.80) **

Note: OR, odds ratio; CI; confidence interval;

[a]Gender, reference male.

[b] Extreme hungry, reference self-reported food deprivation.

[c]Education, reference illiterate.

[d]Marital, reference Married.

[e]Living area, reference rural

[f]BMI, reference normal weight, 18.5≤BMI<24.

** p< 0.01 * p<0.05 ·<0.1

Table S4 Stratified analysis, age 60-85

Variable	Model1 OR (95% CI)	Model2 OR (95% CI)	Model3 OR (95% CI)
Age, per 10 years	1.41 (1.36-1.47) **	1.36 (1.31-1.42) **	1.37 (1.31-1.42) **
Gender[a]	1.94 (1.84-2.05) **	1.66 (1.56-1.76) **	1.70 (1.57-1.84) **
Food deprivation[b]	1.24 (1.17-1.31) **	1.16 (1.09-1.22) **	1.16 (1.1-1.23) **
Education[c]			
Can read and write		0.88 (0.82-0.95) .	0.89 (0.82-0.96)
Primary school		0.71 (0.66-0.77) **	0.71 (0.66-0.76) **
Junior school		0.51 (0.46-0.56) **	0.5 (0.46-0.55) **
High school and above		0.35 (0.31-0.4) **	0.34 (0.3-0.39) **
Marita[d]			
Divorce		1.06 (0.92-1.22)	1.09 (0.95-1.25)
Widowed		1.01 (0.94-1.08)	1.01 (0.94-1.08)
Never married		1.3 (0.95-1.79)	1.26 (0.91-1.73)
Living area[e]		0.63 (0.59-0.66)	0.60 (0.57-0.64)
BMI[f]			
<18.5			1.15 (1.05-1.26)
24-28			1.24 (1.17-1.32) **
≥28			1.65 (1.51-1.81) **
Smoking			1.31 (1.22-1.41) **
drinking			0.71 (0.67-0.76) **

Note: OR, odds ratio; CI; confidence interval;

[a]Gender, reference male.

[b]Food deprivation, reference category none.

[c]Education, reference illiterate.

[d]Marital, reference Married.

[e]Living area, reference rural

[f]BMI, reference normal weight, 18.5≤BMI<24

** p< 0.01 * p<0.05 <0.1

Table S5 Stratified analysis, frailty versus non-frailty and pre-frailty

Variable	Model1<br>OR (95% CI)	Model2<br>OR (95% CI)	Model3<br>OR (95% CI)
Age, per 10 years	1.39 (1.37-1.41) **	1.29 (1.27-1.31) **	1.29 (1.27-1.31) **
Gender[a]	1.64 (1.59-1.68) **	1.43 (1.38-1.47) **	1.48 (1.42-1.54) **
Food deprivation[b]	1.30 (1.26-1.33) **	1.23 (1.19-1.26) **	1.23 (1.19-1.26) **
Education[c]			
Can read and write		0.92 (0.89-0.96) *	0.92 (0.88-0.96) *
Primary school		0.77 (0.74-0.8) **	0.77 (0.74-0.8) **
Junior school		0.63 (0.61-0.66) **	0.63 (0.6-0.65) **
High school and above		0.44 (0.42-0.47) **	0.45 (0.42-0.47) **
Marita[d]			
Divorce		1.21 (1.14-1.29) **	1.23 (1.16-1.31) **
Widowed		1.11 (1.07-1.16) *	1.12 (1.07-1.17) *
Never married		1.48 (1.26-1.75) *	1.46 (1.24-1.72) *
Living area[e]		0.74 (0.72-0.76) *	0.72 (0.7-0.74) **
BMI[f]			
<18.5			1.23 (1.16-1.3) **
24-28			1.09 (1.06-1.13) **
≥28			1.5 (1.44-1.57) **
Smoking			1.26 (1.21-1.31) **
drinking			0.76 (0.73-0.78) **

Note: OR, odds ratio; CI; confidence interval;

[a]Gender, reference male.

[b]Food deprivation, reference category none.

[c]Education, reference illiterate.

[d]Marital, reference Married.

[e]Living area, reference rural

[f]BMI, reference normal weight, 18.5≤BMI<24

** p< 0.01 * p<0.05 <0.1

Table S6 Stratified analysis, men

Variable	Model1<br>OR (95%) CI	Model2<br>OR (95%) CI	Model3<br>OR (95%) CI
Age	1.32 (1.3-1.35) **	1.21 (1.18-1.24) **	1.20 (1.17-1.23) **
Food deprivation[b]	1.30 (1.25-1.35) **	1.22 (1.17-1.27) **	1.21 (1.16-1.26) **
Education[c]			
Can read and write		0.87 (0.81-0.93) *	0.87 (0.82-0.94) *
Primary school		0.74 (0.69-0.79) **	0.74 (0.69-0.79) **
Junior school		0.57 (0.53-0.61) **	0.56 (0.53-0.6) **
High school and above		0.41 (0.38-0.45) **	0.41 (0.37-0.44) **
Marital[d]			
Divorce		1.26 (1.15-1.39) *	1.30 (1.18-1.42) **
Widowed		1.08 (1-1.17)	1.07 (0.99-1.16)
Never married		1.48 (1.25-1.75) *	1.47 (1.24-1.74) *
Living area[e]		0.77 (0.74-0.81) **	0.76 (0.72-0.79) **
BMI[f]			
<18.5			1.21 (1.12-1.31) *
24-28			1.20 (1.15-1.26) **
≥28			1.48 (1.38-1.59) **
Smoking			1.23 (1.17-1.3) **
drinking			0.73 (0.7-0.76) **

Note: OR, odds ratio; CI; confidence interval;

[b]Food deprivation, reference category none.

[c]Education, reference illiterate.

[d]Marital, reference Married.

[e]Living area, reference rural

[f]BMI, reference normal weight, 18.5≤BMI<24.

** p< 0.01 * p<0.05 <0.1

Table S7 Stratified analysis, women

Variable	Model1<br>OR (95%) CI	Model2<br>OR (95%) CI	Model3<br>OR (95%) CI
Age	1.45(1.40-1.50) **	1.36(1.30-1.42) **	1.36 (1.33-1.39) **
Food deprivation[b]	1.29(1.20-1.38) **	1.22(1.14-1.31) **	1.23 (1.19-1.28) **
Education[c]			
Can read and write		0.96(0.87-1.06)	0.95 (0.91-1)
Primary school		0.78(0.71-0.87) **	0.79 (0.75-0.83) **
Junior school		0.69(0.62-0.77) **	0.68 (0.64-0.72) **
High school and above		0.46(0.39-0.55) **	0.47 (0.43-0.52) **
Marital[d]			
Divorce		1.19(1.02-1.39) *	1.21 (1.12-1.31) *
Widowed		1.10(0.99-1.23) .	1.10 (1.05-1.17) .
Never married		1.53(0.58-4.01)	1.46 (0.56-3.84)
Living area[e]		0.71(0.65-0.76) **	0.69 (0.66-0.72) **
BMI[f]			
<18.5			1.25 (1.15-1.35) **
24-28			1.02 (0.98-1.06)
≥28			1.50 (1.42-1.58) **
Smoking			1.30 (1.22-1.38) **
drinking			0.81 (0.75-0.86) **

Note: OR, odds ratio; CI; confidence interval;

[b]Food deprivation, reference category none.

[c]Education, reference illiterate.

[d]Marital, reference Married.

[e]Living area, reference rural

[f]BMI, reference normal weight, 18.5≤BMI<24.

** p< 0.01 * p<0.05 <0.1
